# Very high particulate pollution over northwest India captured by a high-density in situ sensor network

**DOI:** 10.1038/s41598-023-39471-1

**Published:** 2023-08-14

**Authors:** Tanbir Singh, Yutaka Matsumi, Tomoki Nakayama, Sachiko Hayashida, Prabir K. Patra, Natsuko Yasutomi, Mizuo Kajino, Kazuyo Yamaji, Pradeep Khatri, Masayuki Takigawa, Hikaru Araki, Yuki Kurogi, Makoto Kuji, Kanako Muramatsu, Ryoichi Imasu, Anamika Ananda, Ardhi A. Arbain, Khaiwal Ravindra, Sanjeev Bhardwaj, Sahil Kumar, Sahil Mor, Surendra K. Dhaka, A. P. Dimri, Aka Sharma, Narendra Singh, Manpreet S. Bhatti, Rekha Yadav, Kamal Vatta, Suman Mor

**Affiliations:** 1 Research Institute for Humanity and Nature, Kyoto, 6038047 Japan; 2 Institute for Space-Earth Environmental Research, Nagoya University, Nagoya, 4648601 Japan; 3 Faculty of Environmental Science, Nagasaki University, Nagasaki, 8528521 Japan; 4 Research Institute for Global Change, JAMSTEC, Yokohama, 2360001 Japan; 5 Meteorological Research Institute, Japan Meteorological Agency, Ibaraki, 3050052 Japan; 6 Graduate School of Maritime Sciences, Kobe University, Kobe, 6580022 Japan; 7 Center for Atmospheric and Oceanic Studies (CAOS), Graduate School of Science, Tohoku University, Sendai, 9808578 Japan; 8 Faculty of Science, Nara Women’s University, Nara, 6308506 Japan; 9 Atmosphere and Ocean Research Institute, The University of Tokyo, Chiba, 2770882 Japan; 10 Department of Community Medicine and School of Public Health, Postgraduate Institute of Medical Education and Research (PGIMER), Chandigarh, 160012 India; 11 Department of Environment Studies, Panjab University, Chandigarh, 160014 India; 12 Department of Environmental Science Engineering, Guru Jambheshwar University of Science and Technology, Hisar, 125001 India; 13 Radio and Atmospheric Physics Lab, Rajdhani College, University of Delhi, New Delhi, India; 14 School of Environmental Sciences, Jawaharlal Nehru University, New Delhi, 110067 India; 15 Aryabhatta Research Institute of Observational Sciences (ARIES), Manora Peak, Nainital, Uttarakhand 263001 India; 16 Department of Botanical and Environmental Sciences, Guru Nanak Dev University, Amritsar, Punjab 143005 India; 17 Department of Economics and Sociology, Punjab Agricultural University, Ludhiana, Punjab 141004 India

**Keywords:** Environmental sciences, Environmental chemistry, Atmospheric chemistry

## Abstract

Exposure to particulate matter less than 2.5 µm in diameter (PM_2.5_) is a cause of concern in cities and major emission regions of northern India. An intensive field campaign involving the states of Punjab, Haryana and Delhi national capital region (NCR) was conducted in 2022 using 29 Compact and Useful PM_2.5_ Instrument with Gas sensors (CUPI-Gs). Continuous observations show that the PM_2.5_ in the region increased gradually from < 60 µg m^−3^ in 6–10 October to up to 500 µg m^−3^ on 5–9 November, which subsequently decreased to about 100 µg m^−3^ in 20–30 November. Two distinct plumes of PM_2.5_ over 500 µg m^−3^ are tracked from crop residue burning in Punjab to Delhi NCR on 2–3 November and 10–11 November with delays of 1 and 3 days, respectively. Experimental campaign demonstrates the advantages of source region observations to link agricultural waste burning and air pollution at local to regional scales.

## Introduction

Crop residue burning (CRB), occurring immediately after the paddy harvest in the post-monsoon (September–November), is a common practice in Punjab, Haryana and part of Indo-Gangetic Plain (IGP)^1–5^. Emissions from CRB in Punjab not only degrade the air quality at the source but affect the regional air quality of the whole IGP^6–8^. The air quality situation worsens when the airmass reaches directly over the Delhi NCR, a habitat of more than 20 million people. A comprehensive understanding of the emission sources and transport of air pollutions is still lacking due to insufficient measurements from the region, where the remote sensing observations are obscured by clouds or haze.

Combinations of stagnant meteorological conditions and high emissions cause frequent haze events in Delhi NCR^5,9^. The studies reported that during typical haze events, the regional CRB emission contributes up to 78% of PM_2.5_ in Delhi^10–12^. However, accurate quantification is not possible as most of these studies lack in situ observations at the source regions. Further, satellite measurements were obscured by clouds^8^, and model simulation uncertainties arise from emission inventories and chemistry-transport parameterisations^13–16^.

The Delhi NCR tops frequently in the chart of most polluted city/region in the world^17^, having a population-weighted annual average PM_2.5_ of more than 100 µg m^−3^^18,19^. Seasonal load of emissions from CRB elevates the PM_2.5_ pollution in post-monsoon season, coinciding with the festival of lights Diwali. As health risks due to air pollution may rise in Delhi NCR, initiative were taken such as closure of schools/sports events and interruptions in transport systems^5,20,21^. Particulate matter from CRB, 67–90% originated from the agricultural states of IGP, causing around 100 thousand premature deaths every year in India^22^. In addition, there are several other indirect effects of PM_2.5_ pollution on human health, including brain functioning, childbearing, post-neonatal infant mortality, and neurological disorders^23–26^.

This problem of repeated CRB and widespread air pollution in the northwest IGP is a complex and challenging scientific issue to solve. Several national and international projects have been conducted, such as the System of Air Quality and Weather Forecasting And Research (SAFAR), National Clean Air Programme (NCAP), Commission for Air Quality Management in National Capital Region and Adjoining Areas (CAQM), Atmospheric Pollution and Human Health in an Indian Megacity (APHH), and by World Bank and Harvard University to measure and/or evaluate the impact and mitigation of Delhi NCR's severe air pollution in relation with the CRB in Punjab and Haryana^11,27–32^.

The RIHN initiated project AAKASH entitled "An Interdisciplinary Study toward Clean Air, Public Health and Sustainable Agriculture: The Case of Crop Residue Burning in North India", in collaboration with various institutions in India^33^. Here, we aim to elucidate the origin and effects of CRB-related air pollution on Delhi NCR in the post-monsoon season using a dense network of Compact and Useful PM_2.5_ Instrument with Gas sensors (CUPI-Gs). We show the advantages of continuous measurements from a dense network of CUPIs in Punjab, Haryana, and Delhi NCR compared to sparse in situ measurements in the urban area and inadequate satellite data coverage in the presence of clouds.

## Results

### Study area in the NW-IGP and strategy of measurement site network

**Figure 1:**
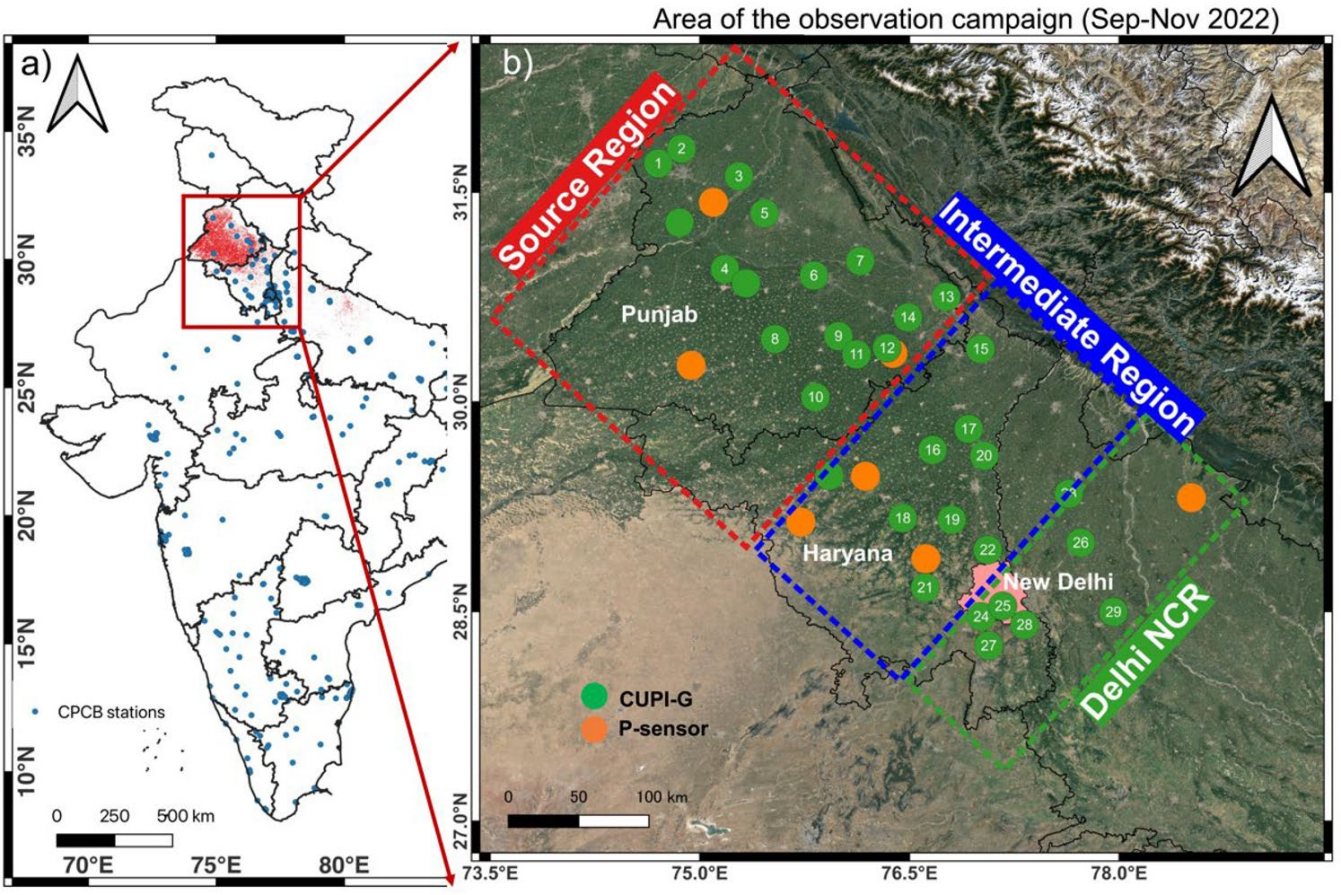
Geographical location of the study area (north-west IGP) of the intensive campaign; the small red dot in left panel (**a**) depicts the VIIRS-based fire counts (FC) in 2022, and blue dots on the map () are the locations of CPCB air quality monitoring stations. In the right panel (**b**), green and orange circles show the location of CUPI-G and P-sensors respectively deployed for air pollutants measurements in a downwind direction in different regions (background depicts the mean landuse-landcover pattern from Google Earth Satellite Imagery). Sites marked by numbers, starting in west Punjab and ending in Delhi NCR, are used in the current analysis. The shapefile for India map is obtained from https://www.aigr.co.in/page/download (last accessed: 22 July 2023) and the plot is generated using QGIS (https://www.qgis.org/en/site/).

The AAKASH intensive measurement campaign of 2022 was conducted using a network of 32 CUPI-G sensors and 7 P-sensors in rural, semi-rural and urban areas of Punjab, Haryana, Delhi NCR and western Uttar Pradesh (Fig. 1). The CUPI-G sensors are equipped with Panasonic PM_2.5_ sensors^34^ and also electrochemical sensors for NO, NO_2_, O_X_(O_3_ + NO_2_) supplied from Alphasense Corp. UK, but the observation results of the electrochemical sensors are not discussed here as the QA/QC and calibration to standard scales are not completed. Out of 32 CUPI-G sites, measurements at 29 sites were conducted without interruption longer than 30 days from 01 September to 30 November 2022 and were selected for this analysis (Table S1; numbered in Fig. 1). The P-Sensors (IHDC Inc., Japan) are equipped with only the Panasonic PM_2.5_ sensors^34^.

Placement of the sensors are made as uniformly as possible along the path of the seasonal winds from northwest to southeast, given the infrastructure available for site access, electricity and mobile communication. Almost all the devices were installed in farmers' houses in farming villages, and locations were selected away from large roads or other sources of pollution by checking Google maps. Referring to the air mass pathways shown by Takigawa and coworkers^8^, we divided the target region into 30 grids and designed the network so that each grid would have at least one site. The existing monitoring stations of the Central Pollution Control Board (CPCB) are located around large cities in the region (Fig. 1). We are the first to establish an air pollution monitoring network in a rural area in a campaign mode. Any bias/prejudice in the site selection would not have achieved the goal of revealing unknowns of air pollution in the CRB source regions and their transport to densely populated urban region.

Members of our partner institutions periodically visited the sites or in the case of system failure, to ensure continuous operation. Based on the prior knowledge of active fire activities, seen as the red dots on the map in Fig. 1, 14 CUPI-Gs (numbered 1 to 14 in Fig. 1) are installed in Punjab, 9 (numbered 15 to 23) are installed in Haryana. The rest of the CUPI-Gs (numbered 24 to 29) are installed in Delhi-NCR. Considering prevalent trade winds and active fire counts, the Punjab region is designated as Source Region, Haryana as the Intermediate region and Delhi NCR as the focus region of the study. The measurements of PM_2.5_ using CUPI are carefully calibrated at Nagasaki University before the field deployment and also after the campaign (see “Methods”). Instrumental health was continuously monitored by comparing our own observations between sites, and in comparison with nearby independent measurements using more sophisticated instrumentation, such as those at the US Embassy, New Delhi and at selected sites operated by CPCB (“Methods” Fig. 7).

### Spatial and temporal variabilities in daily mean PM_2.5_

**Figure 2:**
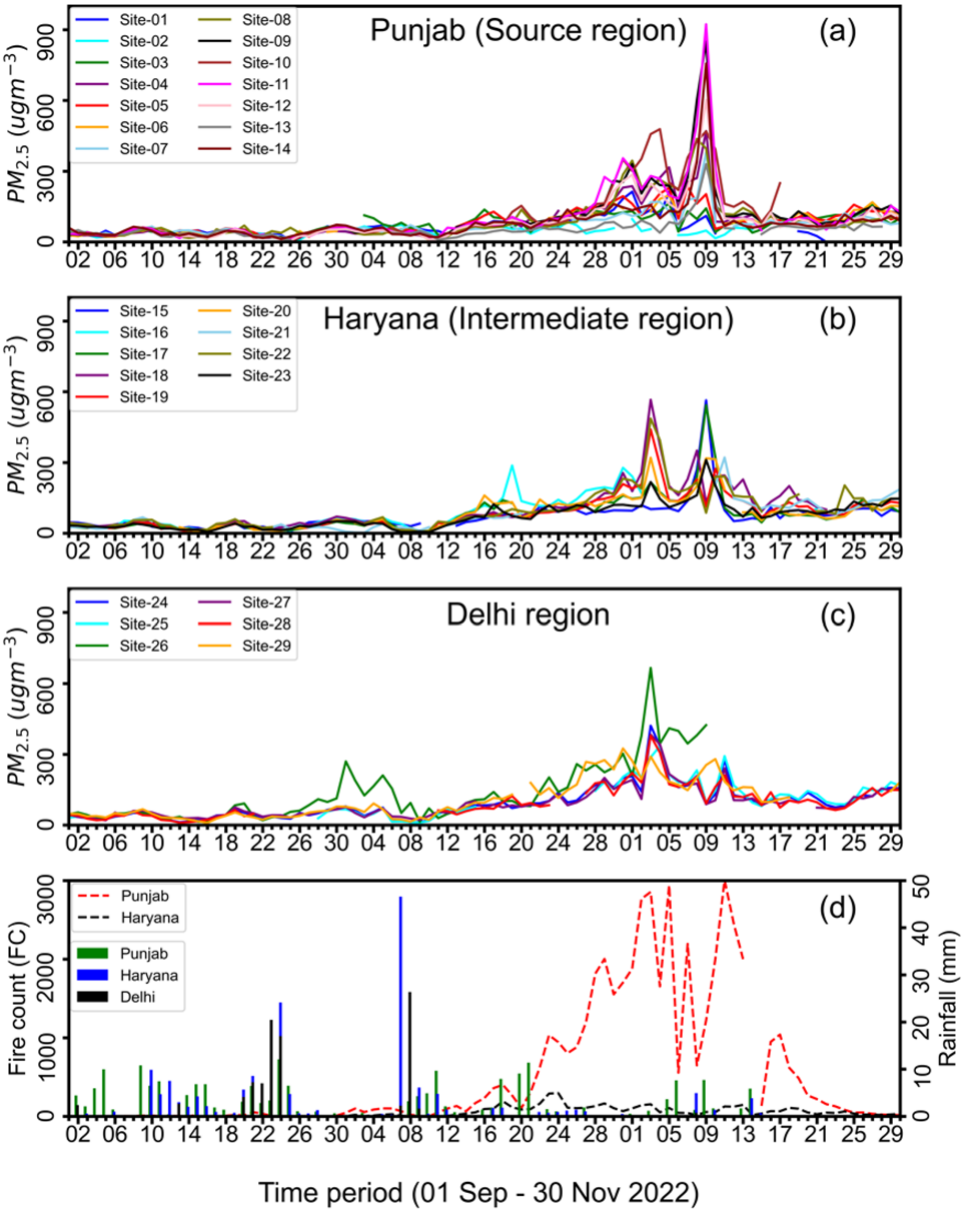
Daily mean PM_2.5_ concentrations over different regions, i.e., Punjab (**a**: source), Haryana (**b**: intermediate) and Delhi NCR (**c**) during the intensive field campaign (1 September–30 November 2022) along with daily VIIRS-based fire counts and over Punjab and Haryana and Global Satellite Mapping of Precipitation (GSMaP) based rainfall (**d**). PM_2.5_ concentrations along with aerosol optical depth (AOD) at individual sites are shown in Fig. S1.

In the post-monsoon season of 2022, active fires of CRB were first detected in the north-west of Punjab (Amritsar region), and fire counts remained low in the rest of the source region (Fig. 2a). The harvesting period encountered frequent rains (Fig. 2d), which led to the delay in harvesting and subsequently shortening the period of the burning of crop residue for next crop cycle. During the campaign period, the daily average concentrations of PM_2.5_ varied widely from day to day; averaging in the range of 80 ± 18 µg m^−3^ at the source sites, 82 ± 11 µg m^−3^ at the intermediate sites, and 104 ± 17 µg m^−3^ at Delhi NCR. The ranges are given as the 1-σ standard deviations of the long-term means at the 29 measurement sites.

All sites show an overall increasing trend in PM_2.5_ concentrations, from a range of 30–40 µg m^−3^ in the late September to monthly means of up to 250 µg m^−3^ for November. Daily mean peak concentrations in November reached 923 µg m^−3^, 566 µg m^−3^ and 666 µg m^−3^ in Punjab, Haryana and Delhi NCR, respectively. This secular increase in concentrations during September–November can be seen in concert with the fire counts in the Punjab region. The daily mean concentrations remained relatively stable at about 150 µg m^−3^ in late October and early November. Note that Indian ambient air quality standards for 24 h is set at 60 µg m^−3^, which exceeded for many 5-day periods in the study region (Fig. S2). The observed daily-mean PM_2.5_ concentration during the CRB period agrees well with the reported values in earlier studies 148.2 ± 20 µg m^−3^ in Punjab and Haryana^35^, 98 ± 1.4 µg m^−3^ in Patiala^36^ and up to 400 µg m^−3^ in Delhi during the peak burning period^8^.

The daily mean concentrations over different regions and peaks of PM_2.5_ concentration are shown in Fig. S1. During the campaign, in the source region, the highest daily mean PM_2.5_ ranges from 97 ± 16 to 923 ± 220 µg m^−3^, and at most of the sites, PM_2.5_ peaked on 09 November. In the intermediate region the highest daily mean ranges from 311 ± 98 to 564 ± 396 µg m^−3^ with peaks on 03 November and 09 November. In Delhi NCR, the highest daily PM_2.5_ varies from 325 ± 102 to 666 ± 459 µg m^−3^, peaks on 03 November except at one site. It is evident from Fig. 2 that the period of 01–15 November observed maximum CRB fire events in Punjab, accompanied by high aerosol load (high PM_2.5_ levels). In addition, we identified two haze events (2–5 November and 8–11 November), the first of which led to the closure of schools by the Delhi government to prevent exposure to severe air pollution^37^; however, the reopening schedule missed information on the forthcoming second peak^38^ due to the lack of real-time observation from the source region.

The cloud cover during the CRB period limited the aerosol (Fig. S3) and fire (Fig. S4) detection capability from the satellites. Figure S3 shows the MODIS Tera/Aqua satellite (MOD/MYD) retrievals of the aerosol optical thickness (AOT; unitless) at local time 10:30 and 13:30 over the CUPI site locations. Both the MODIS satellites missed recording AOT values corresponding to the peak PM_2.5_ at the surface as measured in situ by the CUPI sensors. This is due to mild rainy days and overcast conditions during 5–9 November 2022 (Fig. 2d, Fig. S4). Similarly, the fire detection by VIIRS was also obscured by the cloud cover on the days of highest PM_2.5_ (daily mean exceeding 900 µg m^−3^) on 8–9 November in Punjab. This is the first ever demonstration of very high PM_2.5_ in the fields of CRB, underlining the need for continuous in situ measurement to overcome the data gaps in once a-day overpass of satellites. The MODIS AOT increased by only up to 1.1 times from September to November when averaged over Punjab regions, while the CUPI-G PM_2.5_ increased by more than 4.5 times, suggesting a reassessment of human exposure to PM_2.5_ due to CRB is urgently required.

**Figure 3:**
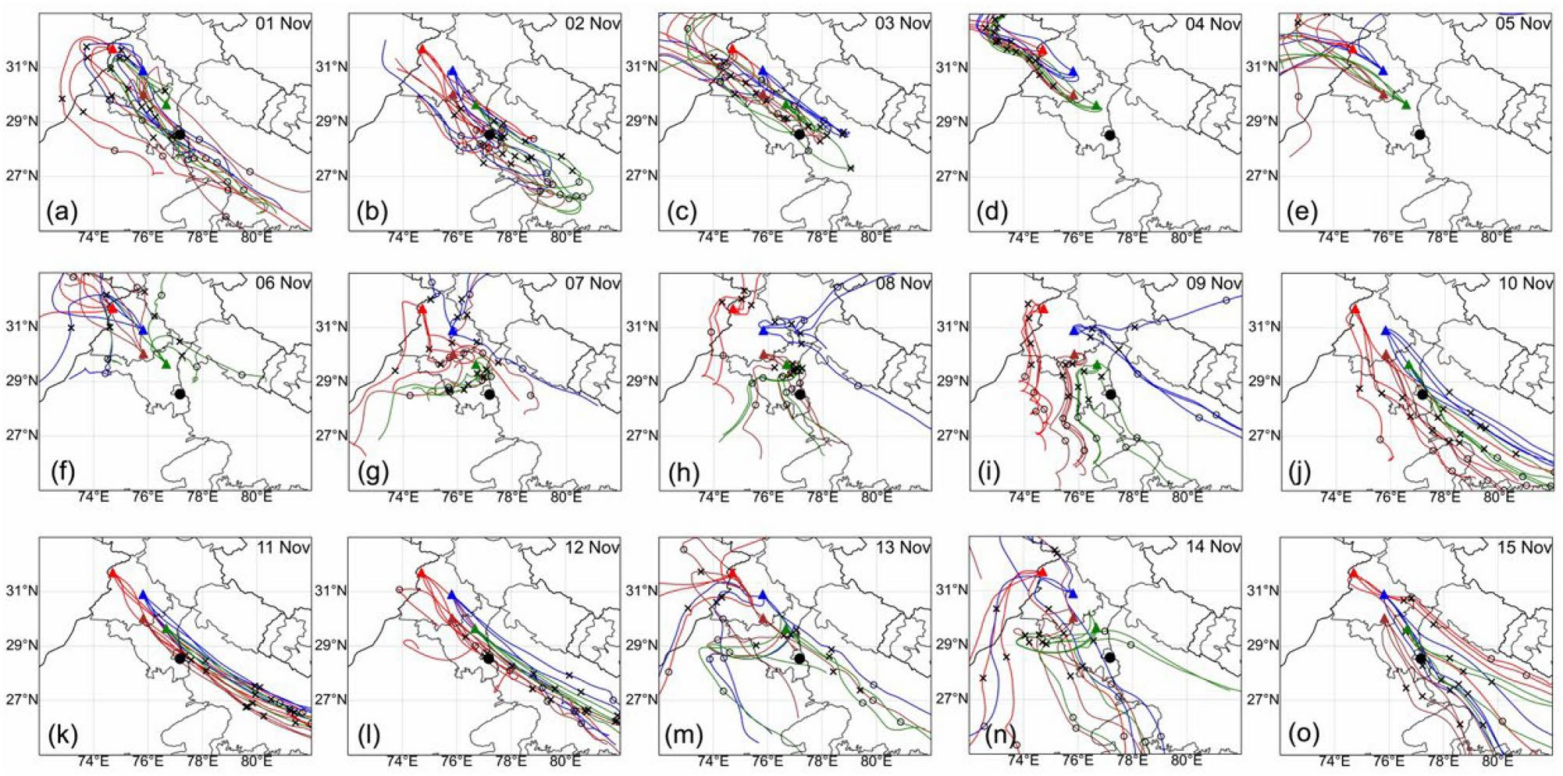
HYSPLIT based 72-h forward trajectories of air mass at 500 m height started at 05:30 IST from four representative locations in source and intermediate region (site# 1: red, 6: blue, 10: dark brown and 16: green) in the source and intermediate region from 01 to 15 November 2022; where the colored triangles show the site location, cross and open circle indicates the location of airmass at 24-h back in time. The black dot shows the location of site 25 (JNU) at Delhi NCR, which is considered as a receptor site. The shapefile for Indian district boundary map is obtained from https://www.aigr.co.in/page/download (last accessed: 22 July 2023) and the plot is generated using QGIS (https://www.qgis.org/en/site/).

To understand the transportation and dispersion of the PM_2.5_ during high PM_2.5_ days (1–15 November), the forward trajectories are calculated using the Hybrid Single-Particle Lagrangian Integrated Trajectory (HYSPLIT)^39,40^ to track the movement of air masses over a 72-h period. The trajectories originated from 4 representative locations in Punjab and Haryana (sites# 1, 6, 10 and 16, seen in red, blue, dark brown and green, respectively, in Fig. 3). We find that the high PM_2.5_ plume observed in Delhi on 3 November is transported directly from the source (Punjab) via the intermediate region (Haryana). The highest PM_2.5_ event of 9 November in Punjab was not transported to Delhi NCR directly but remained stagnated during 7–9 November due to slow wind speeds in Punjab and the trade wind direction changed toward Rajasthan (Fig. 3; Fig. S4). This PM_2.5_ moved to Haryana on 8 or 9 November, depending on site locations and then the airmass reached Delhi NCR on 9 November. The 72-h backward trajectories using HYSPLIT are also calculated and depicted in Fig. S5, which shows the arrival of airmass to Delhi NCR from different directions during 1–15 November.

### Analysis of hourly mean PM_2.5_: diurnal variations and peaks events in Delhi NCR

The diurnal variations in PM_2.5_ for the periods of low burning (01 September to 15 October) and high burning (16 Oct to 30 November) at all 29 CUPI-G sites are shown in Fig. S6. Before 15 Oct, the PM_2.5_ diurnal variations are very low with peak-to-trough amplitudes of about 10 µg m^−3^ and small bimodal peaks can be seen at 7:00 to 10:00 am and 05:00 pm to 08:00 pm, whereas in the period between 16 October and 30 November, U-shaped variations are seen, with the morning peak to afternoon trough as large as 400 µg m^−3^. The meteorology of the planetary boundary layer (PBL) plays a vital role in pollution build-up as the temperature decline in the evening through the night (i.e., shallowing of PBL), leading to an increase in PM_2.5_ concentrations in the presence of emissions on the surface and under the lack of removal mechanism at hourly timescales. The peak-to-trough diurnal cycle amplitudes correlate closely with the daily mean PM_2.5_ concentrations (correlation coefficient, r ranging from 0.35 to 0.91). The PM_2.5_ diurnal cycle also correlated with diurnal change in PBL height, which positively correlated with solar radiation and negatively with vertical temperature gradient and PM_2.5_ levels^41^. In certain meteorological conditions (hot and sunny days), PBL height is more sensitive to solar radiations. The small bimodal peaks during the low burning period can also be related to solid biomass fuel burning used for cooking and heating, which is still prevalent in the IGP region.

**Figure 4:**
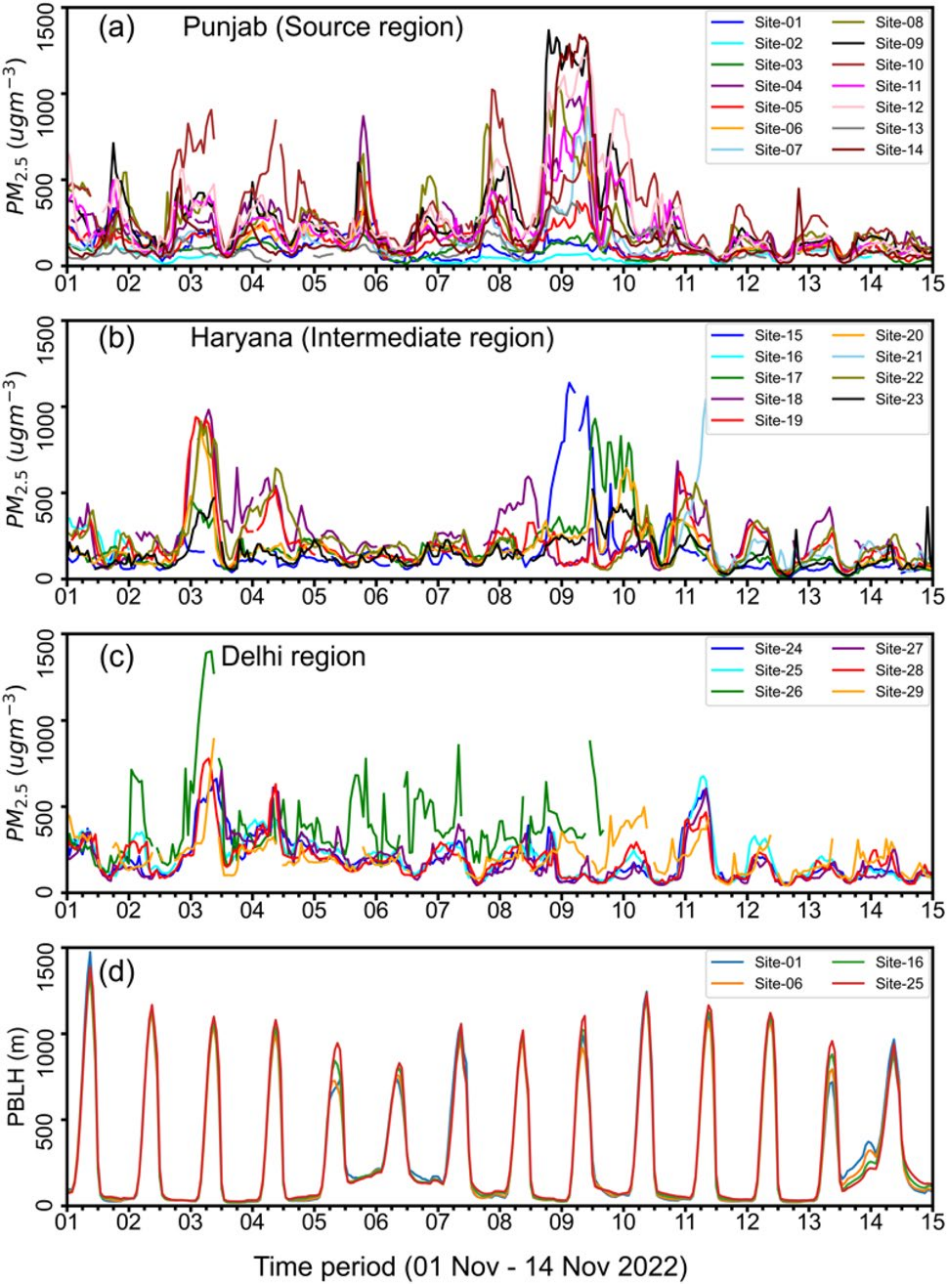
Hourly variations in PM_2.5_ during 01–14 November 2022, i.e., during the period of high CRB over different regions of Punjab (**a**: source), Haryana (**b**. intermediate) and Delhi NCR (**c**). The PBL height from ERA-5 are shown in the bottom panel (**d**).

Figure 4 shows the highest hourly mean PM_2.5_ concentration in the source region, ranging from 140 to 1341 µg m^−3^ and the peak values are almost attained at any time/hour of 8–9 November depending on the sites. Based on our interactions with the farmers/residents, the CRB activities usually occur mid-day when relative humidity is low and straw can catch fire easily. However, we find that most of the PM_2.5_ peaks start building between 15:00 and 18:00 local time and attain daily highs at midnight to early morning, under the influence of the PBL ventilation dynamics (Fig. 4d) in the presence of persistent sources on the surface^42^. The common theories of PBL ventilation are broken on the days of very high emission episodes of 2–3 and 9–10 November at many sites in the Punjab region and at some sites in Haryana, which show sustained high PM_2.5_ values for longer than 24 h (Fig. 4a,b). Prolonged high emissions during the early morning hours through the mid-day maintain supplies of particulates so that the PM_2.5_ lowering effect by the increased PBL ventilation is cancelled. Increase in PBL during the day could also increase PM_2.5_ levels due to intrusion of high PM2.5 from higher altitude (free troposphere), which accumulated by transport from the upwind episodic CRB fire emission.

Examining hourly values reveals characteristics that cannot be captured by the daily average values shown in Fig. 2. First, PM_2.5_ has a large diurnal variation, which is linked to the PBL height. More surprisingly, maximum diurnal values exceeding 1000 µg m^−3^ were often found at many sites in Punjab and in parts of Haryana. Conventional air pollution monitoring networks have focused on monitoring air pollutant concentrations within large cities and have not paid attention to rural areas. However, the fact that the health of rural residents is threatened by air pollution from CRB cannot be ignored.

### Role of meteorology in air pollution build-up and transport of plumes

During the campaign period, there were many rainy days, especially before 15 October which delayed the paddy harvesting and changed the subsequent residue-burning pattern. Air pollution build-up in Delhi NCR from CRB is sensitive to meteorology which changes quickly at around the end of October^5^. Thus, a low impact of a delay of about 10-days in CRB since the groundwater preservation acts in Punjab and Haryana^21,22^ is subjected to further scrutiny. During 2–4 and 9–11 November, Delhi NCR encountered the thick haze event and PM_2.5_ concentration peaked to highest levels, with daily means reaching up to 900 µg m^−3^ and hourly means hovering in the range of 1000–1400 µg m^−3^ at many sites (Figs. 2a, 4a). On high pollution days, the daily mean PM_2.5_ concentration reached 287 µg m^−3^ on 3 November and the average reached 335 µg m^−3^ on 4 November at the JNU site, New Delhi (#25). On 11 November the daily mean PM_2.5_ concentration was 292 µg m^−3^ and the hourly mean peak reached 677 µg m^−3^.

**Figure 5:**
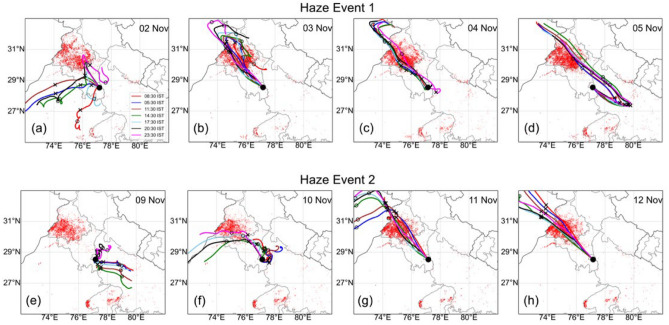
HYSPLIT based 72-h backward trajectories of air masses at 500 m height arriving at Delhi NCR on two haze events (1: top row, on 3 November 2022; 2: bottom row, on 11 November 2022) along with Suomi NPP/VIIRS fire counts (red dots) of the previous 3 days. Fire counts over 3 previous days are shown to avoid gaps in active fire detection due to cloud cover on specific days. The cross and open circle show the location of airmass 24-h and 48-h back in time, respectively. The shapefile for Indian district boundary map is obtained from https://www.aigr.co.in/page/download (last accessed: 22 July 2023) and the plot is generated using QGIS (https://www.qgis.org/en/site/).

The back trajectories, when placed over the fire count distributions (Fig. 5), allow us to understand the buildup of haze events and how the airmass is transported from the source regions over to downwind Delhi NCR through the intermediate region. Transport of high-emission plumes of PM_2.5_ occurs in a narrow swath of passage, which can only be captured by laying a dense observation network of continuous observation.

**Figure 6:**
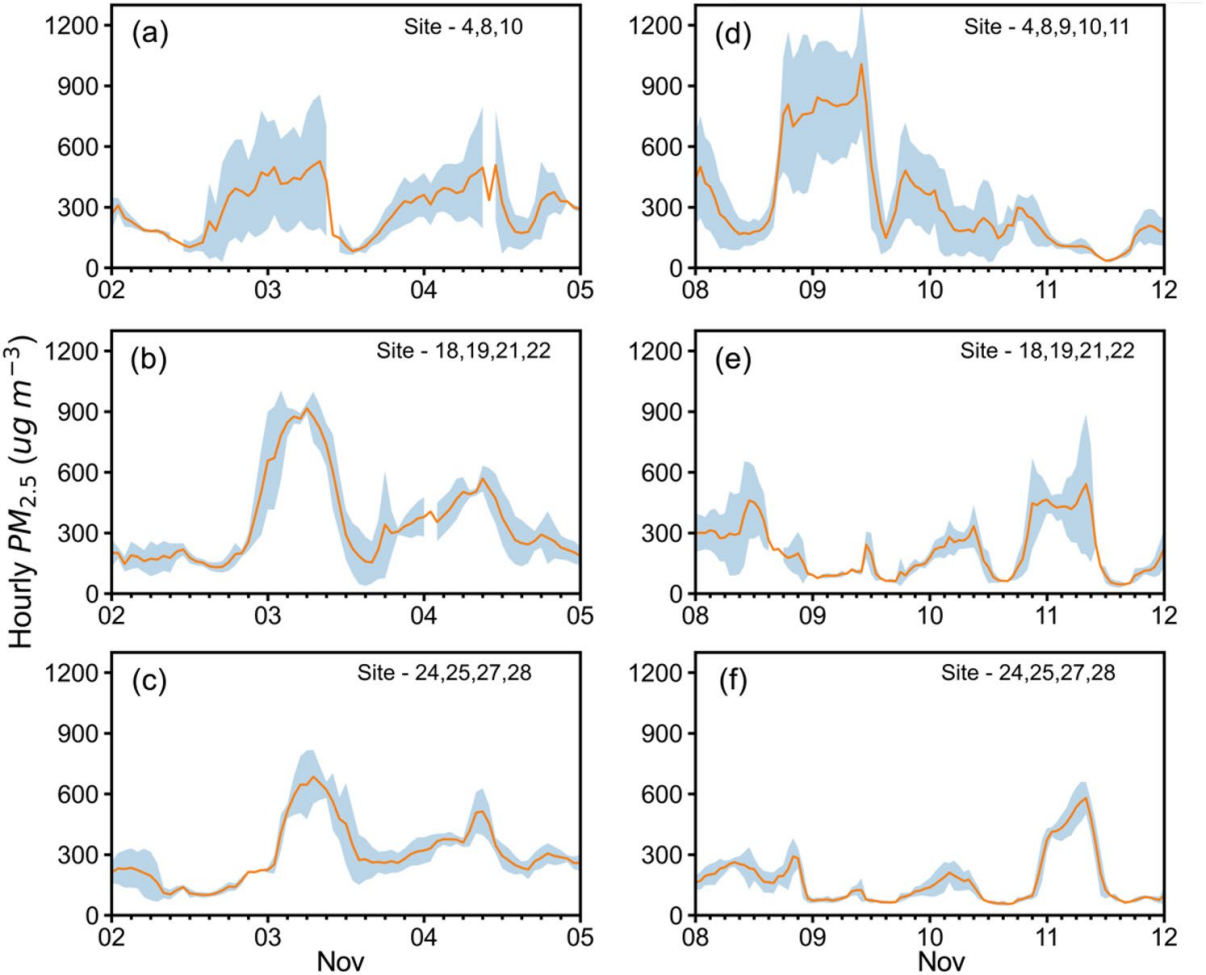
Hourly mean variations in PM_2.5_ along with standard deviation (shaded area) during two haze periods over different regions (**a**,**d**) in Punjab, (**b**,**d**) in Haryana, and (**c**,**f**) in Delhi NCR.

To track the PM_2.5_ plume transport from Punjab to Delhi NCR, we use HYSPLIT simulated 24-h backward trajectories for selecting the sites that are affected by severe CRB cases (ref. Fig. S5). Figure 6 shows the hourly average PM_2.5_ and spread (1-sigma standard deviation) during the two haze periods over these selected sites, separately for the 3 regions. Note that the spreads are as high as or greater than the mean PM_2.5_ values in the source region, but the spreads reduce quickly in the downwind regions as plumes are well organised and restricted to only a few of our CUPI sites only.

Two distinct plumes of PM_2.5_ over 600 µg m^−3^ in Delhi NCR were tracked from Punjab CRB on 2–3 and 10–11 November. For the first haze event, the PM_2.5_ plume was transported within 12–24 h via Haryana, where the concentrations accumulated to as high as 900 µg m^−3^. The second and most widespread haze event in Punjab was delayed by about 72 h before passing over Haryana to Delhi NCR, after a couple of days of dissipation toward Rajasthan (Fig. 3). The severity and spread of the haze can be seen in VIIRS-based true colour images of Earth's surface and the lowermost atmosphere, along with surface wind vectors and the distributions of VIIRS based fire hotspots (Fig. S4). During the peak burning period, the whole IGP was engulfed by a thick haze layer (transparent grey colours), and whenever the winds move towards the IGP from Punjab through Haryana and Delhi NCR, e.g., on 2–5 November 2022 (Fig. S4i–l), and including that of Rajasthan on 9–11 November 2022 (Fig. S4w–y). Separation of contributions of fresh smoke and secondary particulate formation to this widespread haze of IGP sustaining over days requires further investigations using atmospheric chemistry-transport models and additional analyses of the measurements of CO, NO, NO_2_ and O_3_ obtained by our campaign.

## Discussion

The existing air quality network on the ground run by government agencies in India focuses on urban air quality monitoring, where the population density is high. This type of ground network and data gaps in satellite remote sensing due to clouds have failed to capture the linkages between peak CRB emissions in rural areas and the hazardous air pollution events in cities. A dense and strategically positioned measurement network over NW-IGP in September–November 2022 provided a unique and valuable dataset that helped in understanding the impact of crop (paddy) residue burning in the Punjab-Haryana region on the air quality of Delhi NCR and the NW-IGP as a whole. We tested the CUPI-Gs before the campaign for calibration and scaling factors for PM_2.5_ sensors are determined. Our results highlighted that PM_2.5_ has spatial and temporal variabilities. Still, the baseline of PM_2.5_ gradually increased over the NW-IGP in similar manner as that of the fire count increase from late September to the middle of November. Intermittent rainy days delayed the paddy harvesting and CRB activities in September and caused some low PM_2.5_ events.

On two occasions, the daily mean PM_2.5_ exceeded 300 µg m^−3^ in source and intermediate regions and which subsequently reached Delhi NCR with a delay of 1–3 days. The strategic deployment of sensors helped to track the PM_2.5_ peaks at the source, and their transport downwind. The impact of CRB on air quality of the northwest IGP is estimated at factor of 2–4.5, by comparing the daily mean PM_2.5_ values in early September and late October to the middle of November periods. Though most of the CRB activities take place in mid-day, the pollution starts peaking in late afternoon and evening hours and highest in midnight to early morning. Such detailed features in PM_2.5_ variabilities at diurnal, daily, and synoptic time scales are missing from remote sensing satellite measurements by MODIS, VIIRS, particularly for the 2nd haze events of 9–11 November due to cloud cover. The role of chemical transformation (secondary aerosol formation) and chemical characterization of PM_2.5_ must be investigated for clear evidence of the contribution of CRB with the integrations of model simulations.

Continuous measurements in the source region with a strategic array of instruments will enable us to predict severe air pollution events in densely populated Delhi NCR. In addition, directed by the air pollution situation in rural areas, health studies must be conducted to assess exposure to short (days) but extreme air pollution (e.g., daily mean values of reaching up to 900 µg m^−3^).

## Methods

### Ground-based observations and monitoring sites

The intensive measurement campaign was conducted over NW-IGP and the region was divided into 30 grids (~ 60 × 40 km). A network of 32 Compact and Useful PM_2.5_ Instrument with Gas sensors (CUPI-Gs) and 7 P-sensors were deployed in rural, semi-rural and urban areas of Punjab, Haryana, Delhi NCR and western Uttar Pradesh (Fig. 1, Table S1). The sites were selected in the downwind direction of air masses in the post-monsoon season towards Delhi NCR. The CUPI-G grid network was created to cover the CRB source area, intermediate and receptor area downwind for better spatial and temporal coverage. The near real-time measurements were done from 01st September to 30th November 2022.

### Instrumental details

CUPI-Gs are comprised of different sensors and are capable of continuous monitoring of air pollutants including fine particulate matter (PM_2.5_), nitric oxide (NO), nitrogen dioxides (NO_2_), potential ozone (sum of O_3_ and NO_2_) and carbon monoxide (CO) along with temperature (T), humidity (RH), and GPS coordinates. The PM_2.5_ measurements were carried out by a palm-sized optical PM_2.5_ sensor developed by Panasonic^34^, whereas the CO was measured using the CO-B4 Carbon Monoxide Sensor, NO by NO-B4 Nitric Oxide Sensor, NO_2_ by NO_2_-A43F Nitrogen Dioxide Sensor and Ox by OX-A431 Oxidising Gas Sensor. More details about sensitivity, quality assessment (QA), and quality control (QC) can be found at https://www.alphasense.com/. The PM_2.5_ sensor provides the mass concentration based on light scattering measurements. The detailed methodology can be found in Nakayama et al. study^34^. The near-real-time data of every 2 min were generated, stored and transferred to an online server. The data were further processed and cleaned to remove the bias if found.

### Instrument calibration and validation

The calibration and validation of the PM_2.5_ sensors are performed at the laboratories of Nagoya University and Nagasaki University, and the details are available in a published research paper (Nakayama et al., 2018; their Fig. S1). The instruments are intercompared before the infield deployment, and scaling factors for PM_2.5_ sensors are determined for absolute calibration using instrument based on Beta-ray Attenuation Method (BAM). We find a single scaling factor of 1.4 is suitable for all the instruments^43^. The long-term stabilities of sensitivities for a part of PM_2.5_ sensors are also confirmed after recovering the instruments after the intensive measurement period.

**Figure 7:**
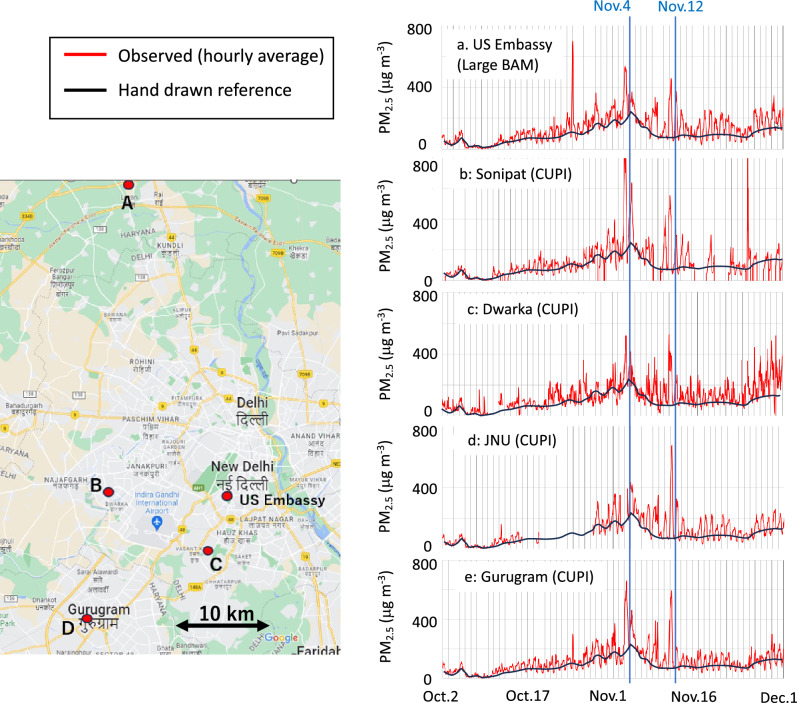
Time series of hourly mean PM_2.5_ concentrations at US Embassy, New Delhi (top right), in comparison with 4 of our sites in the Delhi NCR (red lines). A hand drawn reference line is depicted on each panel for easier comparison of the baselines at different sites (black lines). Location of the sites are marked on the map. The map is taken from https://www.google.com/maps.

There was no periodic calibration of the instrument during the campaign, but we track the health of the instrument by comparing with collocated measurements. An example of PM_2.5_ measured at US Embassy, New Delhi and 3 of Aakash sites and one other PM_2.5_ measurement site (Dwarka) using same type of PM_2.5_ in the neighbourhood. The sites are not perfectly collocated but serves a good purpose of showing excellent co-variability (depicted by the grey line, representing the baseline variation in PM_2.5_ at the US Embassy). In particular, we would like to highlight the agreement of baseline PM_2.5_ concentration at the US Embassy (Fig. 7; grey line), when overlaid on our PM_2.5_ sensor measurements in the Delhi NCR. Individual peaks lasting for less than a few hours are likely effected by local emissions, while the synoptic variability are expected to be regionally representative, such as the peaks during 1–15 November as discussed in the main text.

### Meteorological analysis

The backward and forward trajectory analysis was done by the PC Windows-based HYSPLIT model^40^ using the GDAS 1° meteorological datasets. For wind vectors and PBLH, ERA5 hourly data 0.25° × 0.25° datasets were used^44^. The satellites-based datasets of Visible Infrared Imaging Radiometer Suite (VIIRS) aboard the NASA Suomi National Polar satellite, having a resolution of 375 m and local pass time of 13:30, used for fire detections whereas the AOT datasets were rederived from the MODIS sensor aboard Aqua satellite having a resolution of 3 km and local pass time of 13:30. The meteorological data of rainfall was taken from Global Satellite Mapping of Precipitation (GSMaP) which uses multi-satellite global precipitation map under the Global Precipitation Measurement (GPM) mission.

### Supplementary Information

Supplementary Information.

## Data Availability

All data will be made available, following the acceptance of this article, from the Research Institute for Humanity and Nature website or a permanent data archive.
