# Supplementary material for: Very high particulate pollution over northwest India captured by a high-density in situ sensor network
Source: Sci Rep. 2023 Aug 14;13:13201. doi: 10.1038/s41598-023-39471-1 (PMC10425363; doi:10.1038/s41598-023-39471-1)
Supplement: Supplementary file 1 — Supplementary Information. [file 41598_2023_39471_MOESM1_ESM.pdf]

Supplementary materials for: Very high particulate pollution over northwest India captured by a high-density in situ sensor network

Tanbir Singh[1*], Yutaka Matsumi[2,1*], Tomoki Nakayama[3], Sachiko Hayashida[1*], Prabir K. Patra[4,1*], Natsuko Yasutomi[1], Mizuo Kajino[5], Kazuyo Yamaji[6], Pradeep Khatri[7], Masayuki Takigawa[4], Hikaru Araki[1], Yuki Kurogi[8], Makoto Kuji[8], Kanako Muramatsu[8], Ryoichi Imasu[9], Anamika Ananda[9], Ardhi A. Arbain[9], Ravindra Khaiwal[10], Sanjeev Bhardwaj[11], Sahil Kumar[11], Sahil Mor[12], Surendra K. Dhaka[13], A. P. Dimri[14], Aka Sharma[14], Narendra Singh[15], Manpreet S. Bhatti[16], Rekha Yadav[16], Kamal Vatta[17], Suman Mor[11]

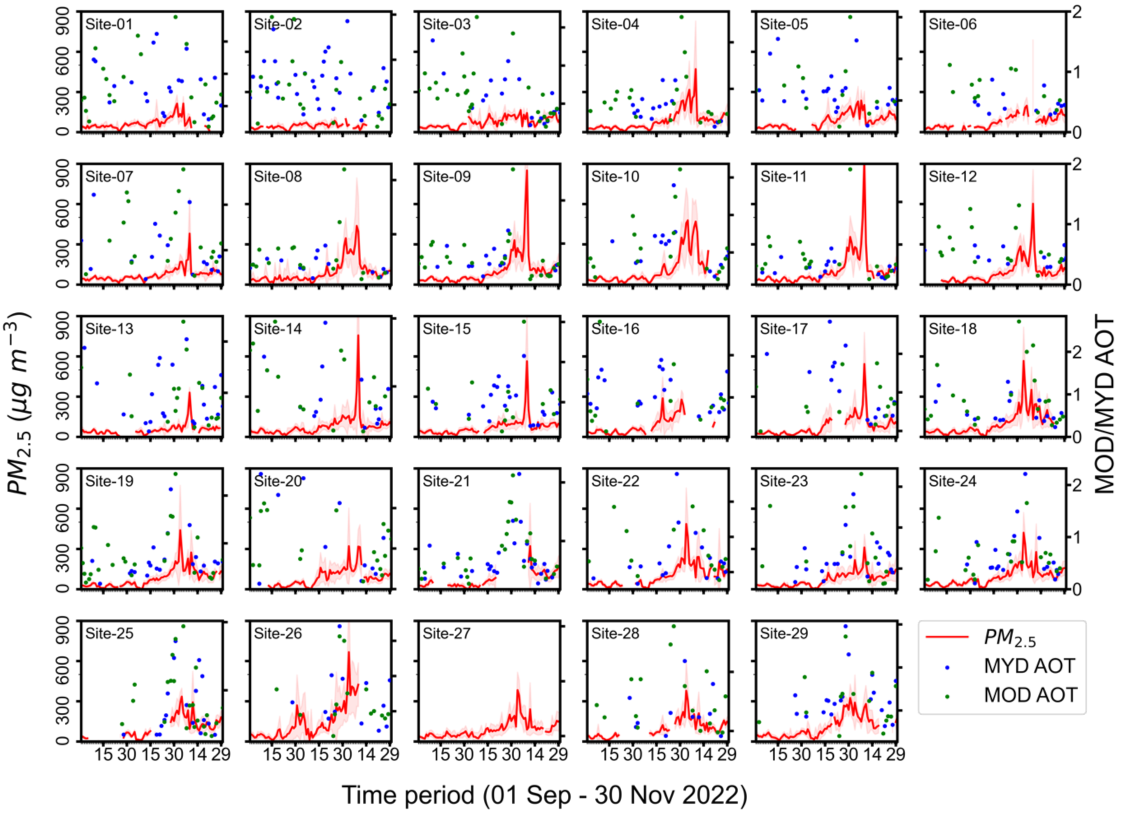

Figure S1: Daily mean $PM_{2.5}$ ($\mu g\ m^{-3}$) concentrations with 1-σ standard deviation (shaded) over all grids of study area between 01st Sept to 30th Nov 2022. For a comparison, we have added the time series of MODIS aerosol optical thickness (AOT) from both Terra (MOD; green dots) and Aqua (MYD; blue dots) satellites. AOTs are sampled at the nearest MODIS measurement location to each of the CUPI-G sites. Note, the high variability in the AOTs limiting detection of peak in aerosol due to crop residue burning.

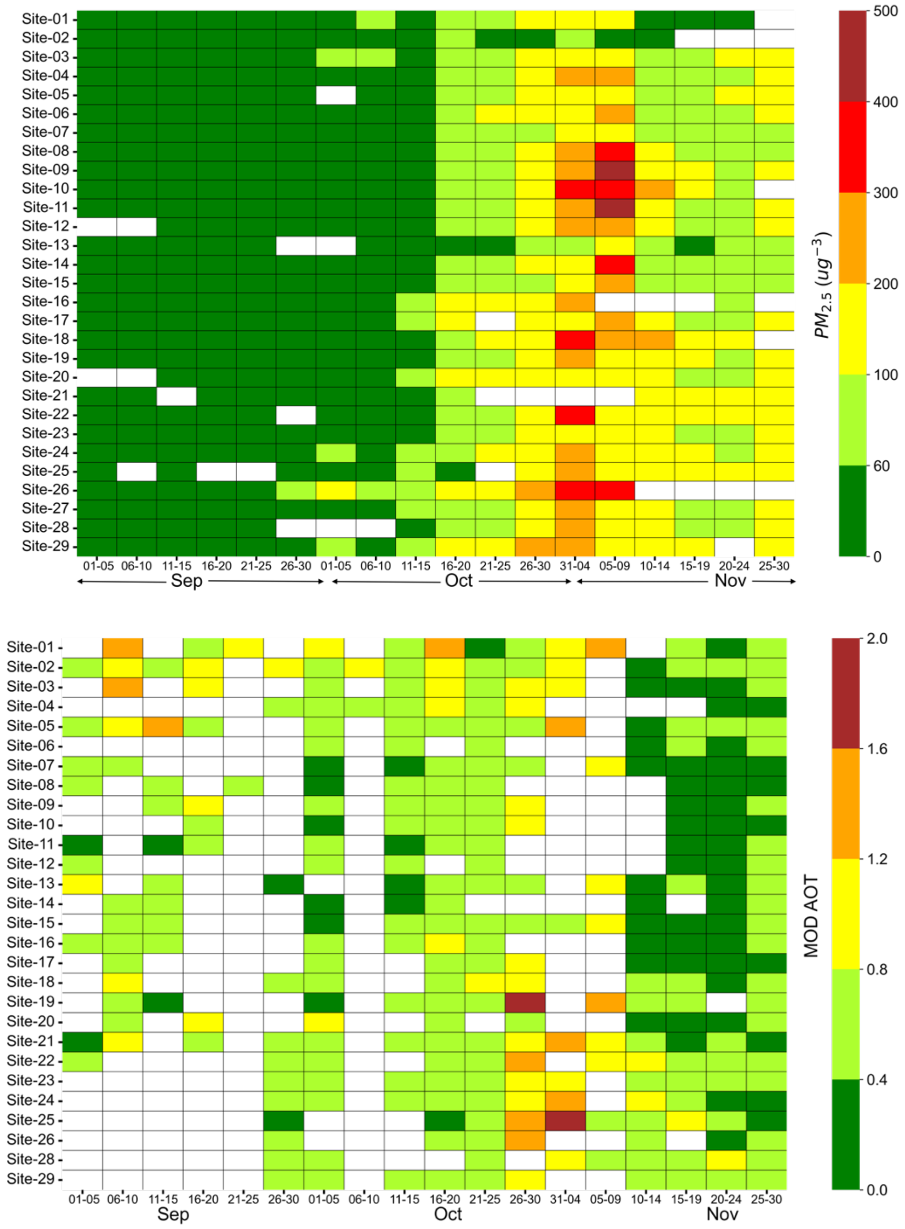

Figure S2: Heatmap of 5-day mean (pentad) PM2.5 concentrations (top panel; a) and MODIS Terra aerosol optical thickness (bottom panel; b) over all sites during the campaign. Note the data gaps in MODIS in the late October and early November when the PM2.5 concentration were high at the surface level. Lack of temporal contrast in AOT during the study period also highlight the lack of sensitivity of MODIS to near surface aerosol concentration. The near surface aerosol concentration is more linked with human exposure.

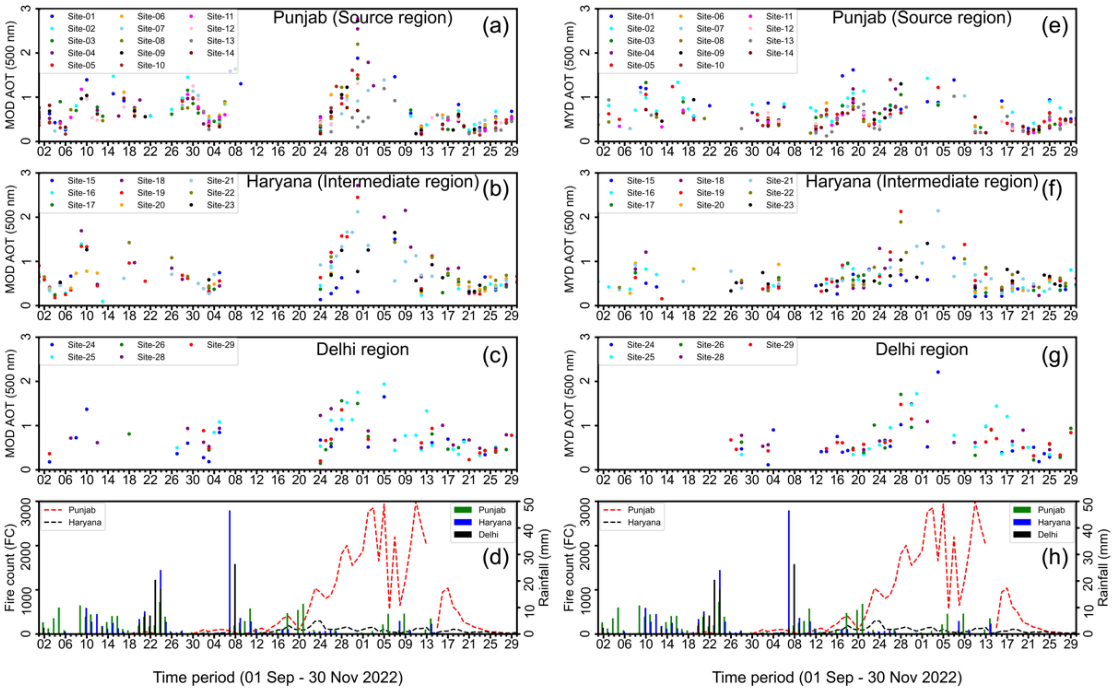

Figure S3: Same as Fig. 2, but for MODIS Tera/Aqua satellite (a-c: MOD/e-g: MYD) based AOT at local time 10:30 and 13:30 over Punjab and Haryana and Delhi NCR.

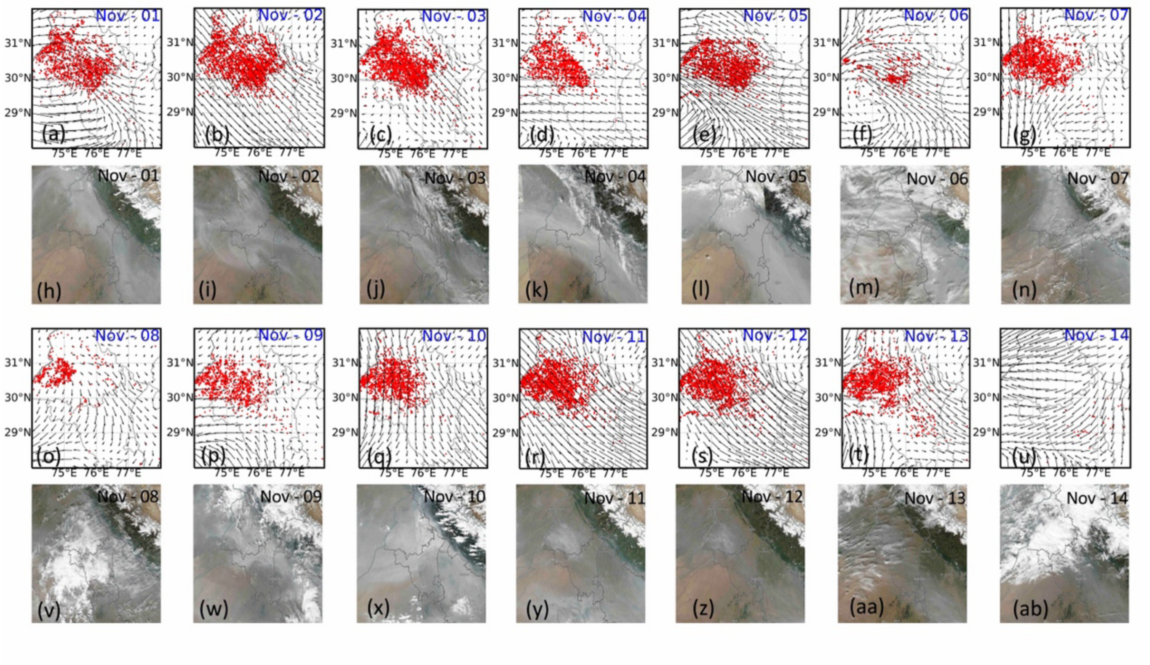

Figure S4: Daily surface wind vectors (in black arrows) along with VIIRS based fire counts (red dots) over the study region (a-g: for the first 7 days of Nov and o-u: for the next 7 days of Nov). The cloud cover maps (pure white) and smoke plumes (transparent grey) are also shown for first (h-n) and next (v-ab) 7 days of November. The cloud cover data are taken from https://wvs.earthdata.nasa.gov/ (VIIRS_SNPP, CorrectedReflectance_TrueColor, Thermal_Anomalies_375m_Day).

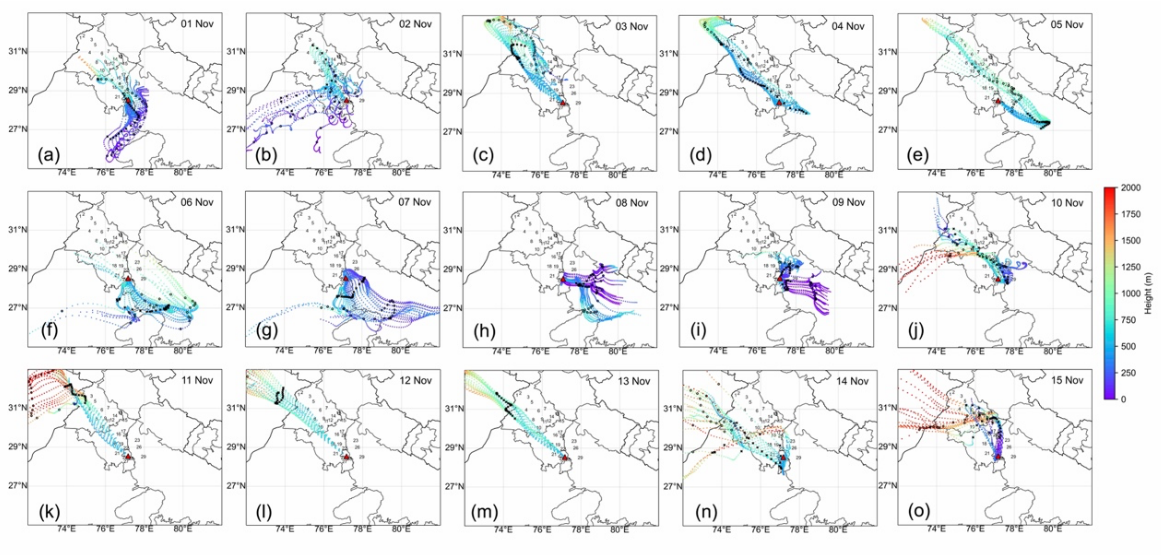

Figure S5: HYSPLIT based 72-h backward trajectories of air masses arriving at Delhi NCR site at different heights from 01-15 Nov. On each panel the locations of our measurement sites are marked so that the plumes of high PM2.5 values can be traced (information used in making Fig. 6).

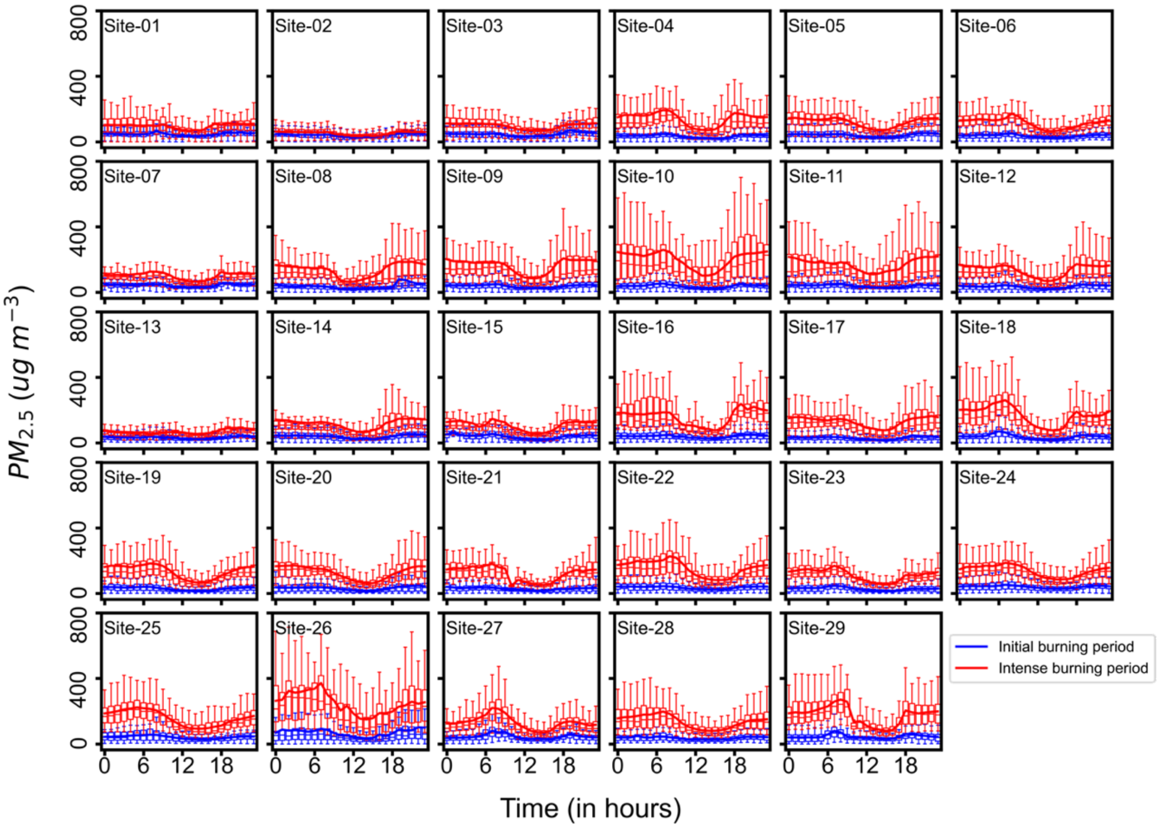

Figure S6: Diurnal variation in $PM_{2.5}$ ($\mu g\ m^{-3}$) during 01 Sep - 15 Oct (initial/pre- burning period) and 16 Oct to 30 Nov (intense burning period) over different regions during the whole campaign.

Table S1: Mean concentrations of $PM_{2.5}$ over different study region and periods during whole campaign.

Site no.	Site name	Latitude	Longitude	Location type	No. of days	01 Sep – 15 Oct 2022	16 Oct – 30 Nov 2022
						$PM_{2.5}$	$PM_{2.5}$
1	Kohali	31.7	74.7	Source	75	46 ± 15	92 ± 50
2	Khatrai Kalan	31.8	74.9	Source	70	41 ± 16	52 ± 18
3	Gaggar Bhana	31.6	75.3	Source	90	48 ± 24	100 ± 30
4	Sadarkot	30.9	75.2	Source	90	34 ± 15	135 ± 82
5	Budo Pandher	31.3	75.5	Source	81	41 ± 18	120 ± 45
6	PAU	30.9	75.8	Source	76	37 ± 14	118 ± 57
7	Rahon	31.0	76.2	Source	90	43 ± 13	100 ± 54
8	Thikriwala	30.5	75.5	Source	91	39 ± 14	139 ± 94
9	Beauscape Farm	30.5	76.0	Source	91	38 ± 15	164 ±140
10	Chajjli	30.0	75.8	Source	80	38 ± 6	201 ±132
11	Nabha	30.3	76.1	Source	88	40 ± 17	168 ±154
12	Patiala	30.4	76.3	Source	80	34 ± 17	136 ± 98
13	Chandigarh	30.7	76.8	Source	77	32 ± 12	72 ± 50
14	Khera	30.6	76.5	Source	91	40 ± 16	117 ±107
15	Karsana	30.4	77.0	Intermediate	88	37 ± 14	106 ± 78
16	Ruksana	29.7	76.7	Intermediate	62	41 ± 20	159 ± 57
17	Taraori	29.8	76.9	Intermediate	83	31 ± 18	131 ± 87
18	Lajwana Kalan	29.2	76.4	Intermediate	83	39 ± 17	169 ± 98
19	Khanpur Kalan	29.2	76.8	Intermediate	89	29 ± 16	134 ± 70
20	Pipalwali	29.6	77.0	Intermediate	78	32 ± 22	129 ± 61
21	Dujana	28.7	76.6	Intermediate	61	29 ± 15	125 ± 54
22	Sonipat	28.9	77.1	Intermediate	81	37 ± 17	154 ± 81
23	Muzaffarnagar (UP)	29.3	77.6	Intermediate	91	28 ± 15	114 ± 44
24	Gurugram	28.5	77.0	Delhi NCR	91	41 ± 20	140 ± 68
25	JNU, New Delhi	28.5	77.2	Delhi NCR	60	40 ± 22	158 ± 63
26	Meerut (U.P.)	29.0	77.7	Delhi NCR	70	70 ± 56	253 ±141
27	Gurugram (Sohna)	28.3	77.1	Delhi NCR	90	47 ± 18	131 ± 67
28	Faridabad(Sector16)	28.4	77.3	Delhi NCR	69	38 ± 17	136 ± 63
29	Aurangabad (UP)	28.5	77.9	Delhi NCR	81	46 ± 21	179 ± 65

Table S2: Five-day mean concentrations of $PM_{2.5}$ over all sites during whole campaign (period September – November 2022).

Site No.	Sep 01-05	Sep 06-10	Sep 11-15	Sep 16-20	Sep 21-25	Sep 25-30	Oct 01-05	Oct 06-10	Oct 11-15	Oct 16-20	Oct 21-25	Oct 25-30	Oct-Nov 31-04	Nov 05-09	Nov 10-14	Nov 15-19	Nov 20-24	Nov 25-29
1	39	44	41	45	28	44	60	61	55	86	68	116	152	104	37	45	21	
2	29	36	39	39	22	41	58	56	46	61	60	52	66	36	43			
3	34	41	42	43	15	47	87	69	60	97	80	121	118	115	62	81	110	114
4	26	38	35	33	16	36	40	32	51	72	93	137	225	291	98	94	78	122
5	32	44	46	46	23	22		42	56	99	91	150	166	186	71	87	107	131
6	28	41	40	36	10	39	38	30	48	76	108	129	151	242	87	93	95	140
7	40	47	44	45	24	44	52	52	39	63	71	95	138	189	74	83	88	94
8	32	39	33	44	40	44	39	36	41	80	75	130	272	320	131	91	69	93
9	30	48	31	39	19	41	48	33	52	79	93	160	260	427	133	107	87	137
10	31	46	29	42	19	44	48	39	46	98	85	166	388	345	204	155	76	
11	34	47	28	38	20	41	54	36	59	85	88	187	287	421	140	78	80	123
12			25	36	16	39	47	30	46	79	84	141	235	297	127	85	72	114
13	39	42	28	37	21			38	22	44	54	63	65	155	93	57	61	64
14	39	49	30	37	19	51	58	30	45	79	92	117	137	303	86	75	78	95
15	37	42	24	30	20	46	56	37	44	73	98	92	103	234	94	85	83	97
16	30	47	26	35	17	46	57	36	76	160	123	179	230				91	
17	24	34	20	26	15	33	45	19	61	125		120	170	244	132	75	71	115
18	36	46	23	36	27	49	54	15	59	76	104	144	326	214	207	145	115	
19	24	37	15	27	16	34	46	10	52	73	88	132	267	156	171	103	89	124
20			16	28	14	38	49	11	60	122	116	130	189	187	169	79	71	109
21	23	48		29	27	25	15	21	49	72					186	115	105	135
22	35	48	22	39	28		51	13	56	86	94	152	305	171	190	124	118	134
23	29	33	17	24	18	38	44	7.8	46	84	93	109	143	169	120	84	92	128
24	29	45	19	45	33	56	62	15	66	84	104	152	261	162	152	107	99	134
25	28		13			49	55	14	61	52		160	252	180	167	111	110	147
26	40	50	25	52	36	93	192	77	67	107	145	243	381	392				
27	31	53	32	48	40	55	57	32	72	79	97	144	241	168	134	96	73	131
28	31	44	16	40	47				59	89	85	155	254	161	149	99	82	131
29	40	53	26	34	32	58	72	23	73	112	146	242	261	192	186	106		123
